# Screening and Identification of Cardioprotective Compounds From Wenxin Keli by Activity Index Approach and *in vivo* Zebrafish Model

**DOI:** 10.3389/fphar.2018.01288

**Published:** 2018-11-13

**Authors:** Hao Liu, Xuechun Chen, Xiaoping Zhao, Buchang Zhao, Ke Qian, Yang Shi, Mirko Baruscotti, Yi Wang

**Affiliations:** ^1^Pharmaceutical Informatics Institute, College of Pharmaceutical Sciences, Zhejiang University, Hangzhou, China; ^2^School of Basic Medical Sciences, Zhejiang Chinese Medical University, Hangzhou, China; ^3^Shandong Danhong Pharmaceutical Co., Ltd., Heze, China; ^4^Department of Bioscienze, The PaceLab, University of Milano, Milan, Italy

**Keywords:** Wenxin Keli, arrhythmia, zebrafish, cardioprotection, drug screen

## Abstract

Wenxin Keli (WXKL) is a widely used Chinese botanical drug for the treatment of arrhythmia, which is consisted of four herbs and amber. In the present study, we analyzed the chemical composition of WXKL using liquid chromatography coupled with high-resolution mass spectrometry (LC-HRMS) to tentatively identify 71 compounds. Through typical separate procession, the total extract of WXKL was divided into fractions for further bioassays. Cardiomyocytes and zebrafish larvae were applied for assessment. *In vivo* arrhythmia model in Cmlc2-GFP transgenic zebrafish was induced by terfenadine, which exhibited obvious reduction of heart rate and occurrence of atrioventricular block. Dynamic beating of heart was recorded by fluorescent microscope and sensitive camera to automatically recognize the rhythm of heartbeat in zebrafish larvae. By integrating the chemical information of WXKL and corresponding bioactivities of these fractions, activity index (AI) of each identified compound was calculated to screen potential active compounds. The results showed that dozens of compounds including ginsenoside Rg_1_, ginsenoside Re, notoginsenoside R_1_, lobetyolin, and lobetyolinin were contributed to cardioprotective effects of WXKL. The anti-arrhythmic activities of five compounds were further validated in larvae model and mature zebrafish by measuring electrocardiogram (ECG). Our findings provide a successful example for rapid discovery of bioactive compounds from traditional Chinese medicine (TCM) by activity index based approach coupled with *in vivo* zebrafish model.

## Introduction

Natural products have played important roles in healthcare system throughout history and will continue to be served as huge and invaluable resource for the discovery of drug candidates. Traditional Chinese Medicine (TCM), widely used in Eastern Asian countries, has been regarded as an important part of natural products for the therapy of various diseases (Wang et al., 2012). The discovery of bioactive constituents from TCM is the key step in the modernization of TCM. As a consequence, developing high throughput methods with satisfied sensitivity for identifying active compounds from complex mixtures of TCM is in great demand. In past two decades, many efforts have been made for rapidly screening of compounds from TCM. Artemisinin (qinghaosu) with antimalarial effect is a successful and impressive example as the gift from TCM (Tu, 2011).

Arrhythmia occurs with abnormal beating of heart myocardium, generally represent disorder of ion channels or cardiomyopathy, can be classified with disorders of impulse formation or conduction. Arrhythmia has intricate pathogenesis, in general, most cardiovascular diseases such as heart failure always accompany with arrhythmia. Some types of arrhythmias are capable of triggering cardiac arrest and sudden death. Unfortunately, most of the antiarrhythmic drugs lack specificity and have numerous adverse effects (Page and Roden, 2005). TCMs have multitargets and synergy effect that benefit with those complex diseases (Li et al., 2011). Wenxin Keli (WXKL) is one of the widely used Chinese patent medicine for arrhythmia and heart failure, and is the first Chinese-developed anti-arrhythmic medicine approved by the China Food and Drug Administration (CFDA) and approved by Chinese Pharmacopeia (ChP), and consists of *Codonopsis pilosula, Polygonatum sibiricum, Radix Notoginseng, Nardostachys jatamansi, Succinum*. Researches in decades have proved that WXKL can suppress and prevent cardiac arrhythmias, including atrial and ventricular arrhythmias (Xing et al., 2013; He et al., 2016; Wang et al., 2016; Li et al., 2017), and inhibit multiple ion channels (Chen et al., 2013; Yang et al., 2017), especially the atrial-selective inhibition of sodium-channel current (Burashnikov et al., 2012), and effect on late Na current (Xue et al., 2013).

Arrhythmia is difficult to model *in vitro*, multiple elements may contribute to the final arrhythmia including genetic predisposition, extrinsic injury, environmental exposures, and stochastic processes. Cell-based model unable to fully reveal the pathological process of arrhythmia. On the other hand, using animal model for screening is costly. Zebrafish (*Danio rerio*) has been a model for biomedical research for decades and is ideal for phenotype-based screen in various ways. The generation time of adult fish is about 3 months and it is easy to maintain a great number of zebrafish with low cost and needn't much space. The embryogenesis can be finished in 24 h post-fertilization (hpf) and each pair of fish can produce more than hundred eggs and the mating is not depended on season. More attractively, their embryos are transparent and most organs including the heart, liver, intestine, and kidney develop in 96 hpf that can be clearly visualized (Barros et al., 2008). The larvae can be manipulated in well-plate and live with a little fluid and is permeable to small molecules (Zhang et al., 2003; Kari et al., 2007). These traits make it possible to establish an easy and high-throughput model to assay the effects of drug candidates on internal organs in the live organism. Zebrafish are easy to genetic manipulation to simulate human disease (Asnani and Peterson, 2014). As a developmental and genetic model, zebrafish has been used for anti-cancer compounds discovery, chemicals toxicity assessment, and so on. Zebrafish heart is highly comparable with human heart in structures, functions, signal pathways, and ion channels (Hu et al., 2000) and is particularly suitable for the study of the cardiovascular system. Here, we used terfenadine, an anti-histamine drug but also a potent hERG blocker and QT prolonger (Dhillon et al., 2013), and was reported that had pro-arrhythmic effects (Chaudhari et al., 2013), to induce the heart disturbance of zebrafish.

**Figure 1:**
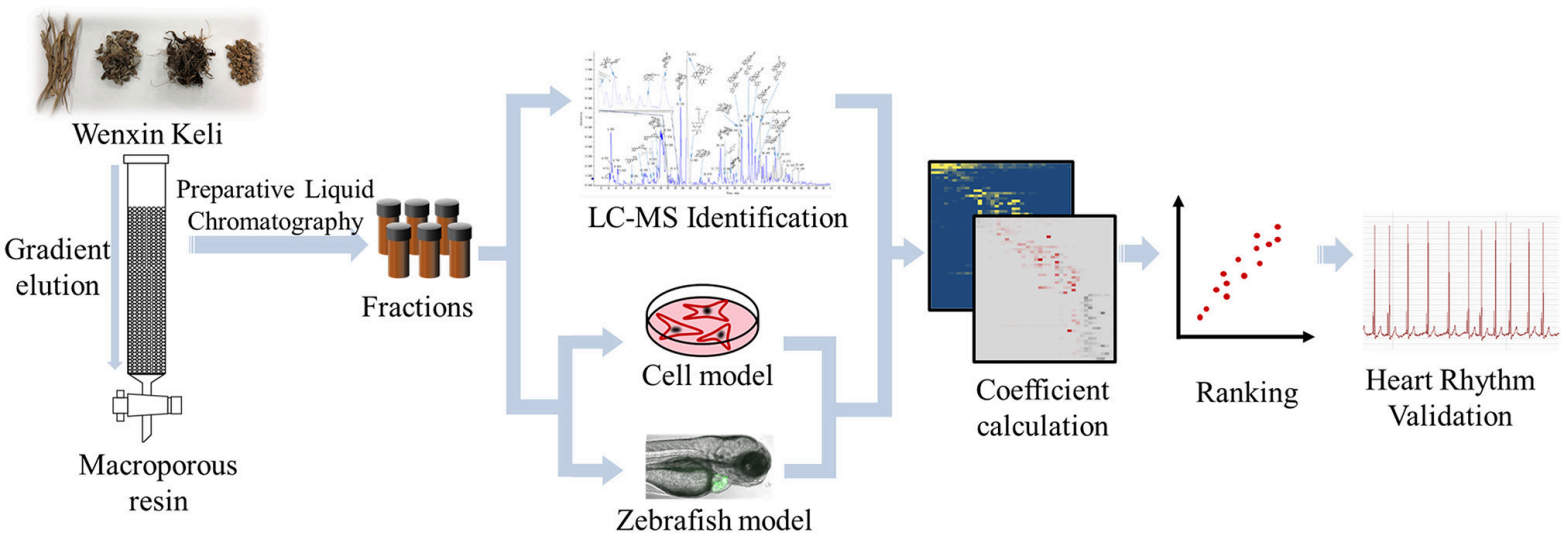
Scheme of active index calculation based active compounds screening from WXKL.

In the present study, we simultaneously used cell-based and zebrafish-based model to assess the cardioprotective and anti-arrhythmia effect of WXKL and the separated fractions, and combined HRMS and chemometric analysis to identify bioactive compounds from WXKL. H9c2 cell damaged by H_2_O_2_ were conducted to evaluate the protective activity of fractions. *In vivo* arrhythmic model based on Cmlc2-GFP transgenic (Tg) zebrafish was applied and the heart rate and rhythm of larvae were measured to evaluate pharmacological effects. The cell viability and heart rate recovery of zebrafish were transformed as the bioactivity coefficient and correlated with the compounds constitute in fractions from WXKL to calculate active index of every compounds. The entire process is illustrated in Figure 1. With both *in vitro* and *in vivo* assessment and active index calculation, several compounds including ginsenoside Rg_1_, ginsenoside Re, notoginsenoside R_1_, lobetyolin, lobetyolinin were selected to validate the activity on larvae and mature fish.

## Materials and methods

### Materials and reagents

Wenxin Keli was obtained from Shanxi Buchang Pharmaceutical Co., Ltd (Shanxi, China). Ginsenoside Rg_1_, Ginsenoside Re, Notoginsenoside R_1_ were purchased from Winherb Medical Tech. Co., Ltd (Shanghai, China). Lobetyolin was obtained from Push Bio-Tech (Chengdu, China). Extract of codonopsis glycosides was obtained from Dasfbio (Nanjing, China).

HPLC-grade acetonitrile and methanol were purchased from Merck (Darmstadt, Germany). Formic acid (HPLC grade) was purchased from Roe Scientific (Newark, DE, USA). Ethanol was purchased from Zhejiang Changqing Chemical (Hangzhou, China). Deionized water was prepared with an Elga PURELAB flex system (ELGA LabWater, UK).

High-glucose Dulbecco's modified Eagle's medium, fetal bovine serum, trypsin-EDTA and antibiotics (100 U/ml penicillin G and 100 g/mL streptomycin) were obtained from Gibico BRL (Grand Island, NY, USA). Tert-Butyl hydroperoxide solution, Thiazolyl Blue Tereazolium Bromide, Terfenadine, DMSO, N-Phenylthiourea (PTU), and Tricaine were acquired from Sigma–Aldrich (St. Louis, MO, USA).

### Apparatus

Tecan infinite M1000 system (Tecan, Zurich, Switzerland). AB TripleTOF 5600^plus^ System (AB SCIEX, Framingham, USA), coupled to a Waters ACQUITY UPLC™ system (Waters, MA, USA). Finnigan LCQ DecaXP^plus^ mass spectrometer equipped with an ESI source (Thermo, MA, USA) coupled to Agilent 1100 liquid chromatography (Agilent, Waldbronn, Germany). Agilent 1200 preparative performance liquid chromatography (Agilent, Waldbronn, Germany). Leica DMI 3000B Fluorescence Inversion Microscope System (Leica Microsystems Inc., USA), Andor Zyla 5.5 sCMOS Cameras (Oxford Instruments plc, Tubney Woods, Abingdon, UK). IX-100F Zebrafish system (iWorx Systems, Inc., USA).

### Characterization of major compounds of WXKL by LC–HRMS

Chemical composition of WXKL was characterized by AB TripleTOF 5600^plus^ System coupled to a Waters Acquity UPLC system. The MS conditions: scan range *m/z* 100–2,000. Negative ion mode: source voltage was−4.5 kV, and the source temperature was 550°C. Positive ion mode: source voltage was +5.5 kV, and the source temperature was 600°C. The pressure of Gas 1 (Air) and Gas 2 (Air) were set to 50 psi. The pressure of Curtain Gas (N_2_) was set to 35 psi. Maximum allowed error was set to ±5 ppm. Declustering potential (DP), 100 V; collision energy (CE), 10 V. For MS/MS acquisition mode, the parameters were almost the same except that the collision energy (CE) was set at 50 ± 20 V, ion release delay (IRD) at 67, ion release width (IRW) at 25. The acquisition parameters for Finnigan LCQ DecaXP^plus^ mass spectrometer were as follows: nebulizing gas, high purity nitrogen (N_2_); collusion gas, high-purity helium (He); ion spray voltage:−3 kV; capillary temperature: 350°C; capillary voltage:−15 V; mass range: *m/z* 100–1,500. Chromatographic separation was carried out on an Agilent Zorbax SB-C18 analytical column (4.6 × 250 mm I.D., 5 μm; Agilent Technologies, USA). The mobile phase consisted of water (A) and acetonitrile (B) both containing 0.05% v/v formic acid. A gradient program was used as follows: 0–5 min, 10% B; 5–15 min, 10–25% B; 15–35 min, 25–35% B; 35–40 min, 35–40% B; 40–45 min, 40–70% B; 45–55 min, 70–95% B; 55–65 min: 95% B. The flow rate was 0.5 mL/min, the column temperature was 30°C, and the injection volume was 20 μL.

### Cell culture and anti-oxidation assays

H9c2 cell were obtained from Cell Bank of the Chinese Academy of Science (Shanghai, China) and cultured in high glucose Dulbecco's modified Eagle's medium supplemented containing 10% fetal bovine serum (FBS) and antibiotics (100 units/mL penicillin and 100 μg/mL streptomycin). The cultures were maintained at 37°C in a humidified atmosphere of 5% CO_2_. The anti-oxidation activity of each fraction was determined by tetrazolium based colorimetric assay (MTT assay). Briefly, cells (5 × 10^4^ cell/mL) were seeded to 96-well plates for 24 h and then treated with fractions of WXKL for another 24 h prior to 150 μM H_2_O_2_ exposure in fresh medium for 3 h, After that, 100 μL 0.5 mg/mL MTT in fresh medium replaced the former medium for 4 h at 37°C. Then, the medium was replaced by 100 μL DMSO and vibrated for 10 min. The cell viabilities of tested fractions were determined by measuring the optimal densities (ODs) of untreated cells (control), the cells exposed to H_2_O_2_ (model), and the cells pre-incubated with components (tested). The activities of the components were calculated using the following formula: Survival rate% = OD of tested/OD of control. Protection rate% = (OD of model – OD of tested)/(OD of model – OD of control).

### Zebrafish husbandry and management

Heterozygotes and homozygote transgenic Cmlc2-GFP zebrafish expressing green fluorescent protein (GFP) exclusively in myocardium were provided by Zebrafish Resource Center, Zhejiang University School of Medicine (Hangzhou, China) and maintained according to established standard procedures. Two parent zebrafish were placed separately in a mating box equipped with a separator to protect the eggs from being eaten. Spawning was induced in the morning and embryos from each box were collected and rinsed with system fish water (containing 0.3% Instant Ocean Salt in deionized water with final pH 6.9–7.2, conductivity 450–550 μs/cm, and hardness of about 90 mg/L NaHCO_3_). The embryos were maintained in the Petri dish with system fish water and transferred to the incubator and incubated at 28°C. This study was granted by the Institutional Animal Care and Use Committee of the Laboratory Animal Center, Zhejiang University. We followed the relevant guidelines from the Laboratory Animal Center of Zhejiang University.

### Zebrafish arrhythmia model and drug incubation

In 24 hpf, larvae with fluorescence were picked under fluorescent microscope and membranes of these larvae were ruptured artificially. Larvae were distributed into a 24-well plate and 8–10 larvae in each well with system fish water added with 0.2 mM N-Phenylthiourea (PTU) and 6 nM methylene blue for treatment. Set groups by wells, including Control, Model, and Treat. Terfenadine was stocked in DMSO at 100 mM, fractions of WXKL was stocked in DMSO at 100 mg/mL and fish water was used to dilute the stock to appropriate concentration. The model group was only given terfenadine, and the treat groups were given terfenadine and corresponding fractions. In 48 hpf incubating, the previous medium was discarded, and added fractions and terfenadine working solution, according to the groups, and filled to 2 mL with fish water medium in each well. The final concentrations of terfenadine was 6 μM. The fractions were diluted to appropriate concentration, mostly 50 μg/mL and some were 25, 12.5, 6.25 μg/mL, depending on the toxicity refer to cell assay.

### Heartbeat recording

In 72 hpf, the beating of zebrafish heart was recorded under florescence with Leica DMI 3000B Fluorescence Inversion Microscope System (Leica). The readout speed of the sCMOS camera was set at 10 frames or 20 frames per second with 4 × 4 pixel binning. L5 filter cube (excitation wave length of 480 nm and emission wave length of 527 nm). One hundred continuous dynamic images were captured by Zyla 5.5 sCMOS Cameras (Andor), subsequently were recognized by Matlab. The area of heart in each picture was measured. The area change with time was supposed to exhibit the heart rhythm. The heartbeats were also recorded manually for accuracy. We calculated the heartbeat of ventricle uniformly.

### Calculation of the activity indexes

The recovery rate (*R*_*i*_) of the components were calculated using the following formula:

$$\begin{array}{l} R_{i}=\frac{B_{i}-B_{M}}{B_{C}-B_{M}}\times 100\% \end{array}$$

*R*_*i*_: normalized heart rate recovery rate of fraction *i*; *B*_*i*_: beats of larvae treated by fraction *i*; *B*_*M*_: beats of larvae treated by terfenadine; *B*_*C*_: beats of larvae treated in control group.

The peak area of each compound was normalized according to the following formula:

$$\begin{array}{l} A_{j}=\frac{A_{i,j}}{\sum_{i=1}^{m}A_{i,j}} \end{array}$$

*A*_*j*_: normalized values of peak area of constituent *j* in fraction *i*; *A*_*i, j*_: peak area of compound *j* in fraction *i*; *m*: the numbers of fractions obtained from whole extract.

The activity indexes of compounds were given by the following formula:

$$\begin{array}{l} AI_{j}=\sum \left(R_{i}{\times A}_{j}\right) \end{array}$$

*AI*_*j*_: activity index of compound *j*.

### Zebrafish ECG measurement

The ECG of zebrafish was measured by IX-100F Zebrafish system (iWorx Systems, Inc., USA). Zebrafish was anesthetic at first and positioned on its back on a fish-bed. Use a paper to gently remove excess water and ensure that the fins are not crossing the belly of the fish. Place the fish-bed with the fish, head to the right, in the chamber and position it under Ag/AgCl surface electrodes. The two electrodes were placed axially along the center-line of the fish's belly and the forward electrode should be placed close to the gills. ECG was recorded by LabScribe v3 software (iWorx System Inc., USA).

### Statistical analysis

The data are expressed as mean ± standard deviation (SD). Parameter comparisons between groups were made with one-way ANOVA analysis of variance. GraphPad prism 7 software (GraphPad Software, USA) was used to carry out statistical analysis. *P* < 0.05 was considered statistically significant.

## Results and discussion

### The chemical composition of wenxin keli extract

**Figure 2:**
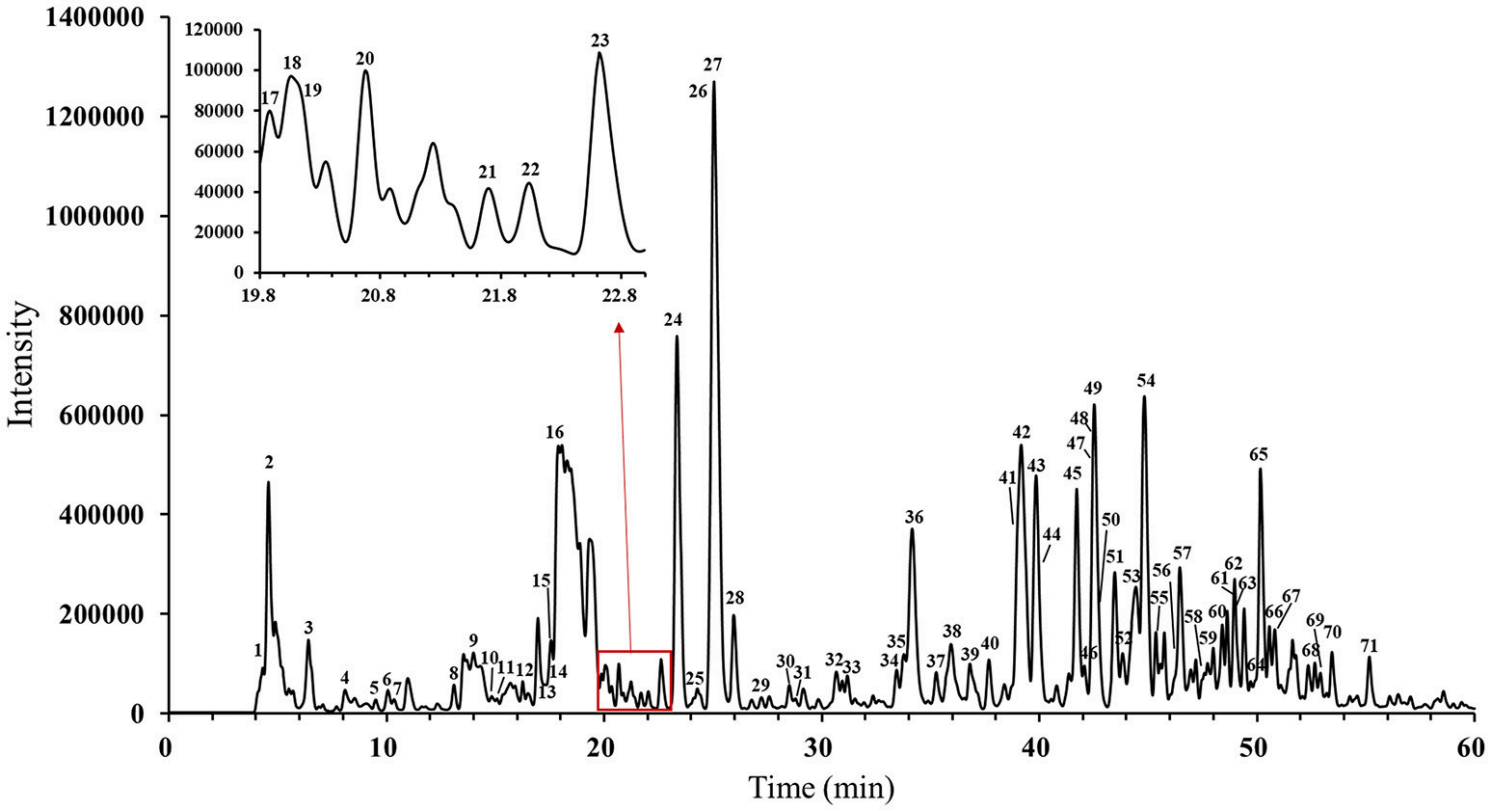
Negative ion model HPLC–MS base peak chromatograms of WXKL.

The main compounds of WXKL include sugar, glycosides, lignans, polyynes, saponins, iridoid glycosides, detailed information is listed in Table 1. The negative ion model base peak LC-MS chromatograms of WXKL was showed as Figure 2. We collected 71 compounds information of MS/MS and identified 53 compounds primarily, including saponins, phenylpropanoids, polyacetylene, triterpenoid, and others. Twenty-seven compounds of them belong to *Notoginseng*, including notoginsenoside R_1_, ginsenoside Rg_1_, ginsenoside Re, ginsenoside Ra_3_, ginsenoside Rb_1_, notoginsenoside R_2_, ginsenoside Rc, ginsenoside Rd and so on. Sixteen compounds belong to *Codonopsis*, including tangshenoside V, lobetyolinin, lobetyolin, atractylenolide III, gentisic acid β-D-glucoside, syringin, hexyl 6-*O*-β-D-glucopyranosyl-β-D-glucopyranoside, hexyl 2-*O*-β-D-glucopyranosyl-beta-D-glucopyranoside and others. Besides, 5 were identified from *Polygonatum*, and 2 were from *Nard* (Table 1).

**Table 1:** Chromatographic and mass spectral data of the constituents of WXKL.

**Peak No**.	**t_R_ (min)**	**Identification**	**Detected (*m/z*)**	**Molecular formula**	**Error (ppm)**	**MS/MS (*m/z*)**	**Source**
1	4.427	Raffinose	503.1598	C_18_H_32_O_16_	−3.9	383.1174	
						221.0651	
						179.0548	
2	4.58	Sucrose or lactose	341.1089	C_12_H_22_O_11_	−1.9	179.0555 [M-H-C_6_H_10_O_5_]^−^	
						161.0459 [M-H-C_6_H_12_O_6_]^−^	
						119.0349 [M-H-C_6_H_10_O_5_-2CH_2_O]^−^	
						101.0247 [M-H-C_6_H_10_O_5_-2CH_2_O-H_2_O]^−^	
3	6.533	Difructose anhydride III	323.0976	C_12_H_20_O_10_	−2.4	99.0459	
4	8.104	Unknown	326.124	C_15_H_21_NO_7_	−1.6	164.0708 [M-H-Glc]^−^	
5	9.508	Vanillic acid 4-*O*-neohesperidoside	475.1446	C_20_H_28_O_13_	−2.3	167.0345 [M-H-Glc-Rha]^−^	HJ
						152.011 [M-H-2C_6_H_11_O_5_]^−^	
						108.0218 [M-H-2C_6_H_11_O_5_-CO_2_]^−^	
6	10.07	Gentisic acid β-D-glucoside	315.0718	C_13_H_16_O_9_	−1.1	153.0182 [M-H-Glc]^−^	DS
						152.0110 [M-H-Glc-H]^−^	
						109.0289 [M-H-Glc-CO_2_]^−^	
						108.0217 [M-H-Glc-H-CO_2_]^−^	
7	10.35	Unknown	375.1287	C_16_H_24_O_10_	−2.6	213.0757 [M-H-Glc]^−^	
						169.0860 [M-H-Glc-CO_2_]^−^	
						151.0752 [M-H-Glc-CO_2_-H_2_O]^−^	
						125.0607 [M-H-Glc-C_3_H_4_O_3_]^−^	
8	13.103	Neochlorogenic acid	353.0876	C_16_H_18_O_9_	−0.6	191.0560 [M-H-C_9_H_6_O_3_]^−^	DS
						179.0344 [M-H-C_7_H_10_O_5_]^−^	
						173.0456 [M-H-C_9_H_8_O_4_]^−^	
						135.0453 [M-H-C_8_H_10_O_7_]^−^	
						107.0503 [M-H-C_10_H_14_O_7_]^−^	
9	14.29	Syringin	371.1339	C_17_H_24_O_9_	−2.3	417.1396 [M-H+FA]^−^	DS
10	15.697	Codonopilate A	718.2728	C_49_H_82_O_3_	*None*	598.2277	DS
		Or Codonopilate B				335.1248	
						303.1002	
11	15.917	Chlorogenic acid	353.0877	C_16_H_18_O_9_	−0.3	191.0557 [M-H-C_9_H_6_O_3_]^−^	DS
						179.0352 [M-H-C_7_H_10_O_5_]^−^	
						173.0449 [M-H-C_9_H_8_O_4_]^−^	
						135.0446 [M-H-C_8_H_10_O_7_]^−^	
12	16.263	Cryptochlorogenic acid	353.0873	C_16_H_18_O_9_	−1.4	191.0560 [M-H-C_9_H_6_O_3_]^−^	DS
						179.0344 [M-H-C_7_H_10_O_5_]^−^	
						173.0456 [M-H-C_9_H_8_O_4_]^−^	
						135.0453 [M-H-C_8_H_10_O_7_]^−^	
						107.0503 [M-H-C_10_H_14_O_7_]^−^	
13	17.038	Unknown	779.2739	C_37_H_48_O_18_	−3.7	437.1571 [M-H-Glc-C_6_H_12_O_6_]^−^	HJ
14	17.529	Vina-ginsenoside R_15_	815.2829	C_33_H_52_O_23_	0.3	861.3002 [M-H+FA]^−^	SQ
						653.4300 [M-H-Glc]^−^	
						491.3754 [M-H-2Glc]^−^	
15	17.562	Unknown	617.2197	C_31_H_38_O_13_	−6.9	663.2250 [M-H+FA]^−^	
						437.1574 [M-H- C_6_H_12_O_6_]^−^	
						365.1357	
						293.1143	
16	17.874	Unknown	455.1662	C_32_H_24_O_3_	2	293.1151 [M-H-Glc]^−^	
17	19.878	Hexyl 6-*O*-β-D-glucopyranosyl-β-D-glucopyranoside	425.2017	C_18_H_34_O_11_	−2.7	471.2076 [M-H+FA]^−^	DS
						179.0560 [M-H-C_12_H_22_O_5_]^−^	
						143.0327 [M-H-C_6_H_14_O-C_6_H_12_O_6_]^−^	
						101.0243 [M-H-C_12_H_20_O_10_]^−^	
18	20.04	Deoxyloganic Acid	359.1342	C_16_H_24_O_9_	−1.5	197.081 [M-H-Glc]^−^	DS or HJ
						153.0917 [M-H-Glc-CO_2_]^−^	
						135.0811 [M-H-Glc-CO_2_-H_2_O]^−^	
19	20.132	Hexyl 2-*O*-β-D-glucopyranosyl-β-D-glucopyranoside	425.2017	C_18_H_34_O_11_	−2.7	263.1492 [M-H-C_6_H_10_O_5_]^−^	
						179.0560 [M-H-C_12_H_22_O_5_]^−^	
						143.0327 [M-H-C_6_H_14_O-C_6_H_12_O_6_]^−^	
						101.0243 [M-H-C_12_H_20_O_10_]^−^	
20	20.678	Tangshenoside V	469.1348	C_21_H_26_O_12_	−0.7	325.0923 [M-H-C_6_H_8_O_4_]^−^	DS
						265.0717 [M-H-C_8_H_12_O_6_]^−^	
						235.0608 [M-H-C_9_H_14_O_7_]^−^	
						205.05 [M-H-C_10_H_16_O_8_]^−^	
						163.0396 [M-H-C_12_H_18_O_9_]^−^	
						145.0289 [M-H-C_12_H_18_O_9_-H_2_O]^−^	
						99.0465 [M-H-C_15_H_18_O_8_-CO_2_]^−^	
21	21.697	Unknown	313.1649	C_16_H_26_O_6_	−2.4	359.1688 [M-H+FA]^−^	
22	22.033	Lobetyolinin	557.2232	C_26_H_38_O_13_	−1.4	603.2292 [M-H+FA]-	DS
						323.0984 [M-H-C_14_H_18_O_3_]^−^	
						233.1166 [M-H-C_12_H_20_O_10_]^−^	
						221.0661 [M-H-C_18_H_24_O_6_]^−^	
						179.0554 [M-H-C_20_H_26_O_7_]^−^	
						161.045 [M-H-C_20_H_28_O_8_]^−^	
						119.0347 [M-H-C_18_H_30_O_12_]^−^	
23	22.621	20-(β-D-glucopyranosyloxy)-ginsenoside Rf or Vina-ginsenoside R_4_	961.5399	C_48_H_82_O_19_	2.2	1007.5456 [M-H+FA]^−^	SQ
						799.493[M-H-Glc]^−^	
						637.4365[M-H-2Glc]^−^	
24	23.362	Notoginsenoside R_1_	931.5281	C_47_H_80_O_18_	1	977.534 [M-H+FA]^−^	SQ
						799.4936 [M-H-Xyl]^−^	
						769.4832 [M-H-Glc]^−^	
						637.4373 [M-H-Xyl-Glc]^−^	
						475.3832 [M-H-Xyl-2Glc]^−^	
25	24.286	Isoheptanol 2(S)-*O*-β-D-xylopyranosyl-(1 → 6)-*O*-β-D-glucopyranoside	409.2071	C_18_H_34_O_10_	−2	276.0881 [M-H-133]^−^	HJ
		Or isoheptanol 2(S)-*O*-β-D-apiofuranosyl-(1 → 6)-*O*-β-D-glucopyranoside				217.0494 [M-H-192]^−^	
		Or n-hexanol *O*-rutinoside					
26	24.923	Ginsenoside Re	945.5443	C_48_H_82_O_18_	1.5	991.5504 [M-H+FA]^−^	SQ
						783.4995 [M-H-Glc]^−^	
						637.4377 [M-H-Glc-Rha]^−^	
27	25.073	Ginsenoside Rg_1_	799.4859	C_42_H_72_O_14_	1.2	845.4913 [M-H+FA]^−^	SQ
						637.4392 [M-H-Glc]^−^	
						475.3822 [M-H-2Glc]^−^	
						391.2879 [M-H-2Glc-C_6_H_12_]^−^	
28	25.963	Lobetyolin	395.1705	C_20_H_28_O_8_	−1.6	441.1765 [M-H+FA]^−^	DS
						233.1180 [M-H-Glc]^−^	
						215.1060 [M-H-Glc-H_2_O]^−^	
						185.0968 [M-H-Glc-H_2_O-CH_2_O]^−^	
						159.0813 [M-H-Glc-H_2_O-C_3_H_4_O]^−^	
						143.0711 [M-H-Glc-C_7_H_6_]^−^	
						125.0603 [M-H-Glc-C_7_H_6_-H_2_O]^−^	
29	27.235	2,2,6-trimethylcyclohexanone	187.0982	C_9_H_16_O_4_	3.3		GS
30	28.512	Unknown	445.0772	C_21_H_18_O_11_	−1	269.0448 [M-H-C_6_H_8_O_6_]^−^	DS or HJ
31	28.796	Notoginsenoside G	959.5233	C_48_H_80_O_19_	1.2	1005.5306 [M-H+FA]^−^	SQ
32	30.678	14-hydroxy-lactarolide A	297.1338	C_15_H_22_O_6_	−1.9	343.1383 [M-H+FA]^−^	
						235.1724 [M-H-62]^−^	
						191.1431 [M-H-106(62+46)]^−^	
33	31.183	Vina-ginsenoside R_2_	827.4801	C_43_H_72_O_15_	0.3	781.4769 [M-H-HCOOH]^−^	SQ
34	33.446	Madecassic acid	503.3373	C_30_H_48_O_6_	−1	459.3116 [M-H-CO_2_]^−^	
		Or terminolic acid					
35	33.772	Unknown	419.1448	C_32_H_20_O	1.6		
36	34.161	Unknown	401.135	C_32_H_18_	3.6		
37	35.266	Notoginsenoside Fa	1239.6392	C_59_H_100_O_27_	1	1285.645 [M-H+FA]^−^	SQ
						1107.603 [M-H- C_5_H_8_O_4_]^−^	
						945.551 [M-H- C_5_H_8_O_4_-Glc]^−^	
						783.4893 [M-H- C_5_H_8_O_4_-2Glc]^−^	
						621.4405 [M-H- C_5_H_8_O_4_-3Glc]^−^	
38	35.949	Unknown	276.0879	C_14_H_15_NO_5_	0.6		
39	36.816	Ginsenoside Ra_3_	1239.6398	C_59_H_100_O_27_	1.5	1285.6455 [M-H+FA]^−^	SQ
						1107.603 [M-H- C_5_H_8_O_4_]^−^	
						945.551 [M-H- C_5_H_8_O_4_-Glc]^−^	
						783.4893 [M-H- C_5_H_8_O_4_-2Glc]^−^	
						621.4405 [M-H- C_5_H_8_O_4_-3Glc]^−^	
40	37.686	Chikusetsusaponin L_5_	901.517	C_46_H_78_O_17_	0.4	947.5234 [M-H+FA]^−^	SQ
		Or Chikusetsusaponin LM_2_				769.4828 [M-H-132]^−^	
41	38.845	Notoginsenoside R_4_	1239.6381	C_59_H_100_O_27_	0.1	1285.645 [M-H+FA]^−^	SQ
						1107.603 [M-H- C_5_H_8_O_4_]^−^	
						945.551 [M-H- C_5_H_8_O_4_Glc]^−^	
						783.4893 [M-H- C_5_H_8_O_4_-2Glc]^−^	
						621.4405 [M-H- C_5_H_8_O_4_-3Glc]^−^	
42	39.161	Ginsenoside Rb_1_	1107.5963	C_54_H_92_O_23_	0.6	1153.6012 [M-H+FA]^−^	SQ
						1061.5274 [M-H-46]^−^	
						945.554 [M-H-Glc]^−^	
43	39.855	Notoginsenoside R_2_	769.4742	C_41_H_70_O_13_	−0.2	815.4801 [M-H+FA]^−^	SQ
						637.4367 [M-H-Xyl]^−^	
						619.4250 [M-H-Xyl-H_2_O]^−^	
						475.3813 [M-H-Xyl-Glc]^−^	
						391.2869 [M-H-Xyl-Glc-C_6_H_12_]^−^	
44	39.923	Polygonatoside D or isomer	899.465	C_45_H_72_O_18_	0.5	753.2310 [M-H-Rha]^−^	HJ
						737.4188 [M-H-Glc]^−^	
						429.2190 [M-H-2Glc-Rha]^−^	
45	41.725	S-ginsenoside Rg_2_	783.4894	C_42_H_72_O_13_	−0.8	829.4958 [M-H+FA]^−^	SQ
						637.4348 [M-H-Rha]^−^	
						475.3809 [M-H-Rha-Glc]^−^	
46	42.073	Ginsenoside Rc	1077.585	C_53_H_90_O_22_	−0.1	1123.5909 [M-H+FA]^−^	SQ
						945.5531 [M-H- C_5_H_8_O_4_]^−^	
						783.4964 [M-H- C_5_H_8_O_4_-Glc]^−^	
						621.4409 [M-H- C_5_H_8_O_4_-2Glc]^−^	
						459.3871 [M-H- C_5_H_8_O_4_-3Glc]^−^	
47	42.147	R-ginsenoside Rg_2_	783.4896	C_42_H_72_O_13_	−0.5		SQ
48	42.422	Ginsenoside Rb_2_	1077.585	C_53_H_90_O_22_	−0.1	1123.5909 [M-H+FA]^−^	SQ
49	42.533	(20R)-Ginsenoside Rh_1_	637.4309	C_36_H_62_O_9_	−1.9	683.4371 [M-H+FA]^−^	SQ
						475.3793 [M-H-Glc]^−^	
50	43.06	Ginsenoside Rb_3_	1077.585	C_53_H_90_O_22_	−0.1	1123.5909 [M-H+FA]^−^	SQ
51	43.469	Ginsenoside Rh_1_	637.4308	C_36_H_62_O_9_	−2.1	683.4374 [M-H+FA]^−^	SQ
						475.3793 [M-H-Glc]^−^	
52	43.835	Unknown	489.3205	C_29_H_46_O_6_	−3.4	535.3265 [M-H+FA]^−^	
53	44.41	5,6,9-trihydroxy-octadec-7-enoic acid	329.2332	C_18_H_34_O_5_	−0.4	229.1443	DS
						211.1338	
54	44.788	Ginsenoside Rd	945.5423	C_48_H_82_O_18_	−0.6	991.5476 [M-H+FA]^−^	SQ
						783.4963 [M-H-Glc]^−^	
						621.4396 [M-H-2Glc]^−^	
55	45.359	Ginsenoside F_1_	637.4308	C_36_H_62_O_9_	−2.1	683.4374 [M-H+FA]^−^	SQ
						475.3793 [M-H-Glc]^−^	
56	46.429	Unknown	677.2456	C_33_H_42_O_15_	0.7		
57	46.468	Unknown	501.3214	C_30_H_46_O_6_	−1.5	547.3264 [M-H+FA]^−^	
58	47.455	Notoginsenside T_5_	751.4631	C_41_H_68_O_12_	−0.9	797.4692 [M-H+FA]^−^	DS
						619.4254 [M-H- C_5_H_8_O_4_]^−^	
59	47.995	Aractylenolide III	247.1343	C_15_H_20_O_3_	1.3		DS
60	48.41	Unknown	487.3042	C_29_H_44_O_6_	−4.7	533.3104 [M-H+FA]^−^	
						441.3025 [M-H-46]^−^	
61	48.933	20(S)-Ginsenoside Rg_3_	783.4897	C_42_H_72_O_13_	−0.4	829.4962 [M-H+FA]^−^	SQ
						621.4415 [M-H-Glc]^−^	
						459.3859 [M-H-2Glc]^−^	
62	48.975	Ginsenoside Rh_4_	619.4192	C_36_H_60_O_8_	−3.8	665.4272 [M-H+FA]^−^	SQ
		Or Ginsenoside Rk_3_					
63	49.098	20(R)-Ginsenoside Rg_3_	783.4895	C_42_H_72_O_13_	−0.7	829.4962 [M-H+FA]^−^	SQ
						621.4415 [M-H-Glc]^−^	
						459.3856 [M-H-2Glc]^−^	
64	49.71	3-(4′-hydroxy-benzyl)-5,7-dihydroxy-6,8-dimethyl-chroman-4-one	313.1078	C_18_H_18_O_5_	−1.1	207.0649 [M-H-C_7_H_6_O]^−^	HJ
						205.0502 [M-H-C_7_H_8_O]^−^	
						179.0700 [M-H-C_9_H_10_O]^−^	
						165.0549 [M-H-C_9_H_8_O_2_]^−^	
65	50.172	Unknown	485.326	C_30_H_46_O_5_	−2.6	531.3322 [M-H+FA]^−^	
						441.3002 [M-H-CO_2_]^−^	
66	50.573	Lobetyol	247.1342	C_14_H_18_O	0.9	247.1342 [M-H+FA]^−^	DS
67	50.819	Nardosinone	249.1497	C_15_H_22_O_3_	0.3	295.1550 [M-H+FA]^−^	GS
68	52.662	Unknown	457.2949	C_28_H_42_O_5_	−2.3	295.2452 [M-H-Glc]^−^	
69	52.921	Ginsenoside Rk_1_	765.4785	C_42_H_70_O_12_	−1.2	811.4851 [M-H+FA]^−^	SQ
		Or Ginsenoside Rg_5_				603.4289 [M-H-Glc]^−^	
						279.1597 [M-H-3Glc]^−^	
70	53.449	Unknown	455.2793	C_28_H_40_O_5_	−2.2	501.2858 [M-H+FA]^−^	
						411.2912 [M-H-CO_2_]^−^	
71	55.169	Unknown	499.3056	C_30_H_44_O_6_	−1.8	545.3107 [M-H+FA]^−^	
						455.3182 [M-H-CO_2_]^−^	
						411.3280 [M-H-2CO_2_]^−^	

*DS, Codonopsis pilosula; HJ, Polygonatum sibiricum; SQ, Radix Notoginseng, GS, Nardostachys jatamansi*.

### Evaluating cardioprotective effect of components by zebrafish arrhythmia model

We first performed standard isolation by preparative chromatography to obtain fractions, which were analyzed by Finnigan LCQ DecaXP^plus^ mass spectrometer. The mass spectrums of every fractions were shown in Supplementary Material.

Oxidative stress plays a key role in the pathogenesis of various diseases (Furukawa et al., 2004). These fractions were then evaluated the protective activity on H9c2 cell damaged by H_2_O_2_. The toxicity of all fractions were tested at first, and the safe concentration of most fraction were 50 μg/mL, and some were 25/12.5/6.25 μg/mL to insure no toxicity. The cell viability with these fractions treated were shown in Supplementary Material.

Zebrafish (*D. rerio*) has been an ideal model for drug screening (Goldsmith, 2004). There have been many applications of zebrafish as a high-throughput screening model and cardiotoxicity risk assessment of drug candidates (Wen et al., 2012; Zhu et al., 2014). Here we used Cmlc2-GFP Tg zebrafish as the base. This transgenic line expressing GFP exclusively in myocardium driven by promoter cmlc2 (cardiac myosin light chain 2 gene) (Huang et al., 2003). Terfenadine has been reported can induce QT prolongation in zebrafish and guinea pig (Milan et al., 2006; Lu et al., 2012; Chaudhari et al., 2013), which is associated with ventricular tachyarrhythmia (Gowda et al., 2004). Terfenadine causes QT prolongation in adult zebrafish, also demonstrate in zebrafish embryos (Langheinrich et al., 2003).

**Figure 3:**
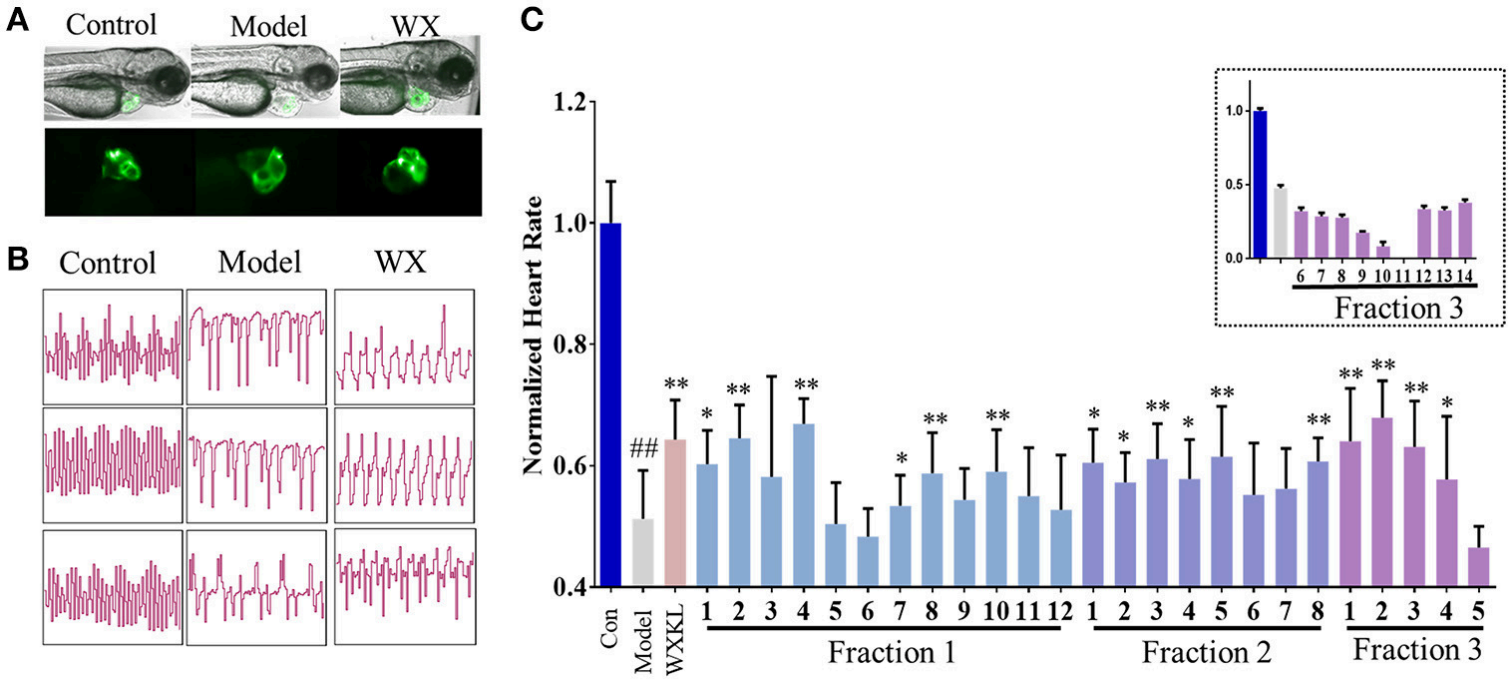
Cardioprotection of Wenxin Keli and fractions on zebrafish model. **(A)** Represent images of heart structures in zebrafish under bright-field and fluorescence. **(B)** Represent images of heartbeat rhythm of different groups exhibited by Matlab. **(C)** Normalized heart rate of zebrafish larvae influenced by WXKL fractions. *n* ≥ 8. ^##^*P* < 0.01 vs. control, *P < 0.05, **P < 0.01 vs. model.

As shown in Figure 3, terfenadine had less effect on the structure of the heart (Figure 3A) but influenced the rhythm of beat obviously (Figure 3B). The rhythm of heartbeat was exhibited through the area change of heart analyzed by Matlab. It's obvious that the control groups performed fast and regular rhythm (about 180 beats/min), and in the model groups, the heart rate were down to 80–100 beats/min with irregular heartbeats after incubated with terfenadine for 24 h, and some showed typically atrioventricular block (Peal et al., 2011), while co-incubated with WXKL stabilized the rhythm (Figure 3B, Supplementary Videos). And WXKL and its fractions performed varying affection on heart rate. Fraction 1 and Fraction 2 and former part of Fraction 3 showed beneficial effect on heart rate, while the remaining parts of Fraction 3 lowered the rate even more (Figure 3C).

### Screening active compounds by activity indexes calculation and ranking

**Figure 4:**
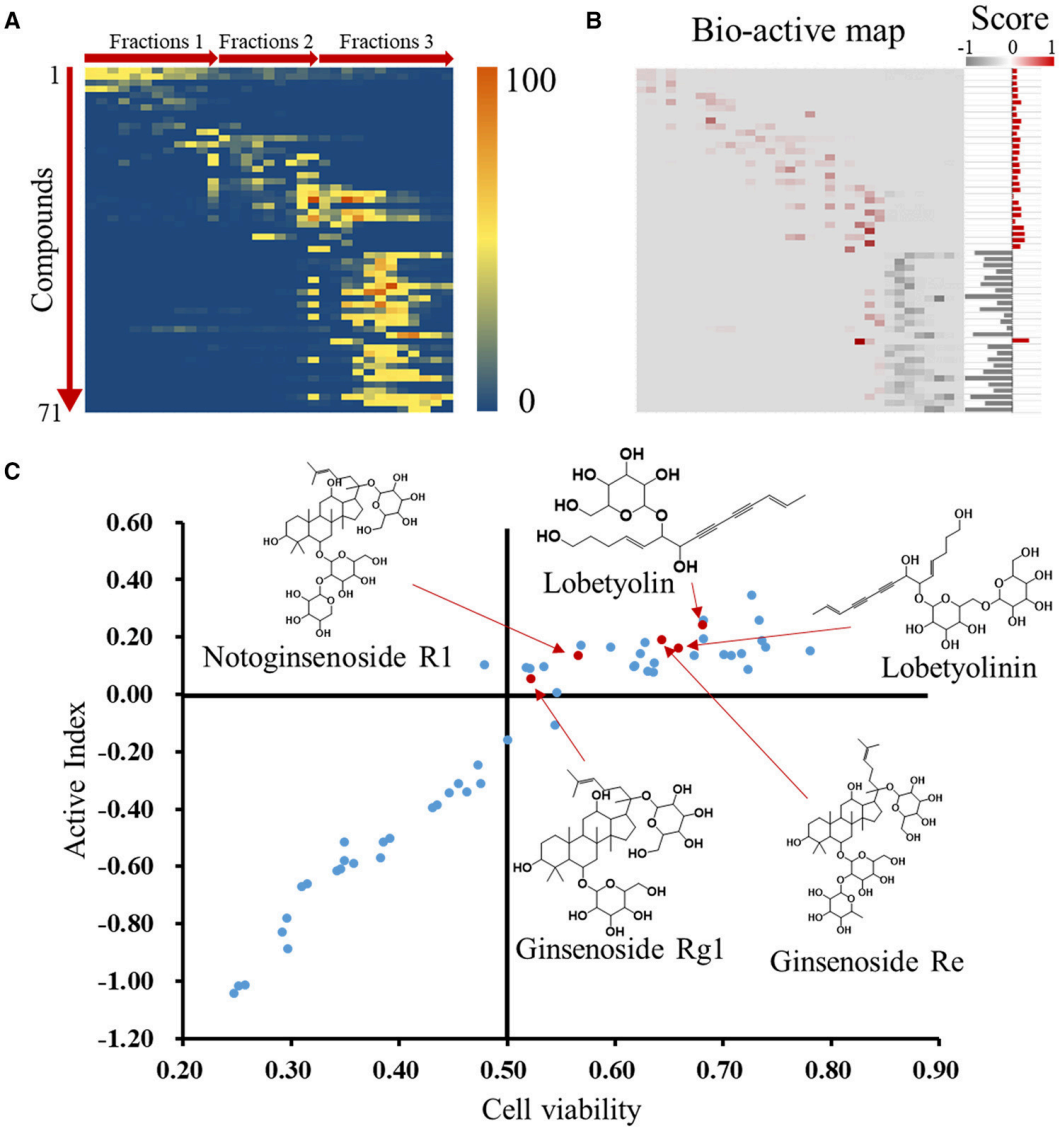
Identification of active compounds by coefficient ranking. **(A)** Heatmap for relative content of identified compounds in each WXKL fractions. **(B)** Bio-active map was converted from content map and corresponding bioactivity coefficient in each fraction, and active indexes of identified compounds were calculated and showed on the right. **(C)** Compounds were plot by cell protection and bio-active scores and mass spectrum of several compounds on top. X axis means the contribution of compounds to the cardiomyocytes protection. Y axis means the *AI* of compounds.

Activity index (*AI*) of each compound was calculated according to mathematical formulae proposed in our previous study. It was assumed that the compounds with positive activity index might be active and has contribution to the activity of whole formula to some extent (Wang et al., 2014). The relative intensities of the identified compounds in each fraction were visually presented in a heatmap (Figure 4A). After multiply corresponding bioactivity coefficient (i.e., heart rate recovery rate) of each fraction, the heatmap was converted into a bio-active map, and the red and gray color represent good or bad effect, respectively. The calculated scores were exhibited as histogram on the right (Figure 4B). The detailed scores were listed in Supplementary Material. We plot compounds with the effect on cardiomyocytes and the heart rate of zebrafish (Figure 4C), the compounds in upper right region represented a better activity.

### Validation of active compounds

**Figure 5:**
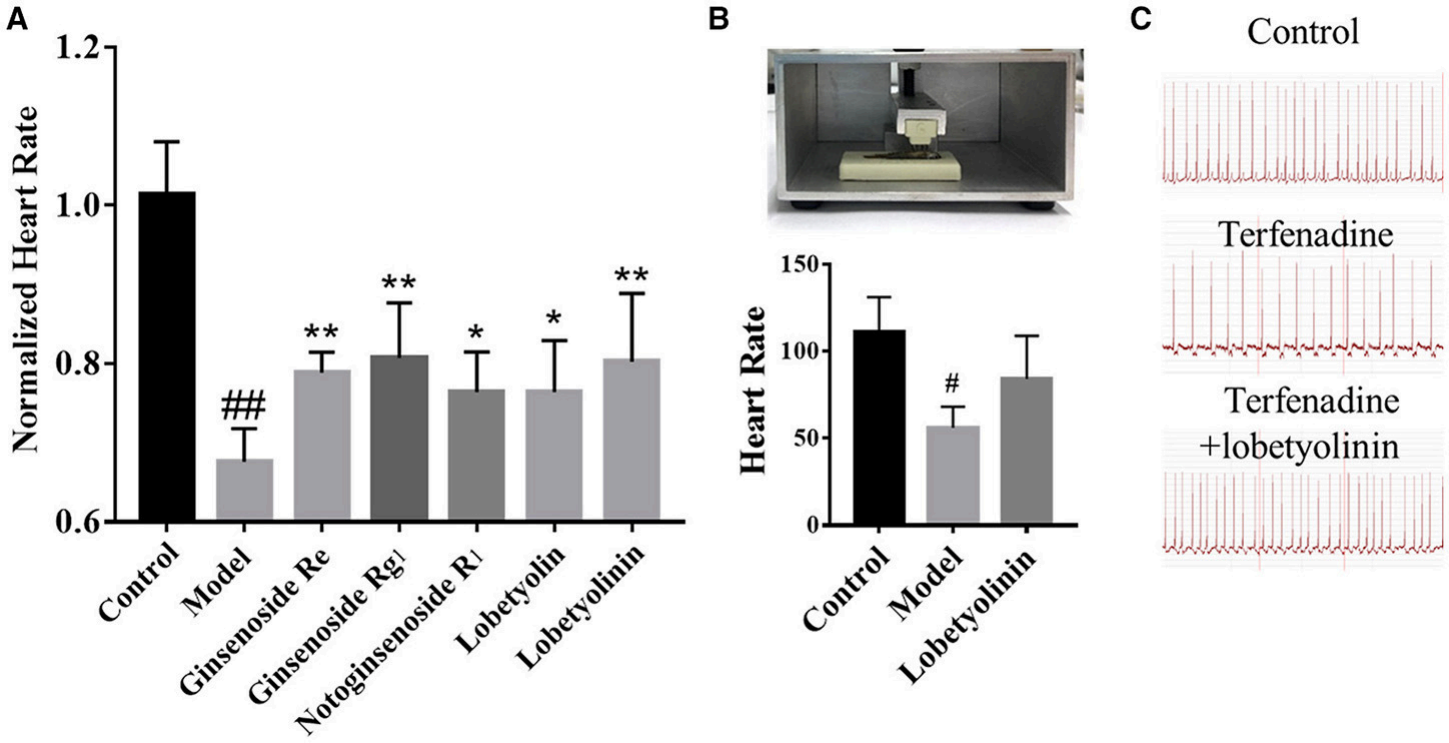
**(A)** Normalized heart rate of zebrafish larvae treated with compounds. *n* = 7. ^#^*P* < 0.05 vs. control, ^*##*^*P* < 0.01 vs. control, *P < 0.05, **P < 0.01 vs. model. **(B)** The schematic of ECG measurement, and the result of different group. *n* = 3. **(C)** Represent electrocardiograms of adult zebrafish treated in different groups.

According to the scores, Ginsenoside Rg_1_, Ginsenoside Re, Notoginsenoside R_1_, Lobetyolin, Lobetyolinin were selected to validate activity, considering the available. Lobetyolinin was prepared and enriched by ourselves from commercial codonopsis glycosides. Their toxicity was confirmed before. We increased the dosage of terfenadine and shortened the incubation time as an acute injury model to improve significance when validating the active of pure compounds by reason of the pure compounds were not strong enough to exhibit activity in original method. After pre-treated with compounds (50 μM) for 24 h, the larvae were treated with 15 μM terfenadine for 2 h and recorded heartbeat under fluorescent. As the consequence, the heart rate of larvae was recovered in varying degree (Figure 5A). Ginsenoside Rg_1_ and lobetyolinin exhibited better activities. Meanwhile, ECG of adult zebrafish treated with compound was measured. The heart rate of normal zebrafish was around 100 beats/min, and after treated with 25 μM terfenadine for 1 h, the heart rate was down and occurred irregular rhythm (Figure 5B). Lobetyolinin pre-treated for 6 h recovered the heart rate and represent electrocardiograms were showed as Figure 5C.

## Discussion

A few researches described the chemical components of WXKL. Wang et al. established a database for the chemical components of the five herbs in WXKL for active compounds predication (Wang et al., 2017), however, all the compounds were acquired from database refer to the herb not the real composition of the patent drug, and the chemical components probably change during the manufactory process. We analyzed the extract of WXKL directly with LC-HRMS at beginning, but the analytic method we established still has limitation. Actually, the compounds in Fraction 2 weren't separated clearly and seemed have low mass spectrum response, which make this portion of fractions have similar composition. Compounds identified from *Nard* and *Succinum* were rare, maybe for the reason that the main constitutes of *Nard* and *Succinum* are volatile oil, which are more appropriate analyzed by gas chromatography–mass spectrometry (GC–MS). According to report, the extracts of *Nard* significantly blocked *I*_*Na*_ and *I*_*Ito*_ of rat ventricular myocytes (Liu et al., 2009). The active compounds we predicted especially on the top are minor composition were difficult to get standard substance for bioactive assays except ginsenoside Re, ginsenoside Rg_1_ and notoginsenoside R_1_, which limited the further validation. We attempted to isolate substances such as lobetyolinin from extracts of *C. pilosula*. It has reported that ginsenoside Re has negative effect on cardiac contractility and autorhythmicity (Peng et al., 2012), ginsenoside Rg_1_ prolonged ventricular refractoriness and repolarization (Wu et al., 1995), notoginsenoside R_1_ has protective effects on cardiovascular system (Li et al., 2014). Related activity of lobetyolin has few reports.

Drug-induced model is a common approach, verapamil and terfenadine were applied to develop a zebrafish heart failure model (Zhu et al., 2018). QT prolonging is a typical characteristic of arrhythmia which also can be induced by cisapride and astemizole besides terfenadine (Langheinrich et al., 2003). It has to be considered that drug treated by oral may cause unstable effect, so design rational approaches of drug treatment is necessary. Chaudhari et al. performed parenteral administration of terfenadine with different doses and recorded ECG to assess drug-induced QTc prolongation in zebrafish. Those with the doses above 1 mg/kg were observed some proarrhythmic effects such as Ventricular Premature Contractions, Ventricular Tachycardia, Atrio-Ventricular (AV) Block, and Torsade de pointes (TdP) (Chaudhari et al., 2013). However, the cardiotoxic of terfenadine is possible not associated with QT prolongation and the occurrence of TdPs, but with marked widening of the QRS complex and other cardiac arrhythmias (Hondeghem et al., 2011). It's reported that terfenadine caused non-TdP like VT/VF by slowing of conduction via blockade of I_Na_ (Lu et al., 2012). In addition, transgenic zebrafish lines are also feasible to avoid the unstable results of drug induce method. Several mutants were identified that exhibited arrhythmias (Milan and Macrae, 2008) like the bradycardic line *slo mo*, with variable degrees of sinoatrial or atrioventricular heart block (Baker et al., 1997). Besides, some mutants exhibited recessive lethal phenotypes included mutants such as *tremblor* (Langenbacher et al., 2005), *island beat* (Rottbauer et al., 2001), and *reggae* (Hassel et al., 2008). The Tg line that express fluorescent proteins (e.g., Cmlc2-GFP) was beneficial for optical measurement from the other perspective.

ECG (electrocardiogram) abnormalities is the critical characteristic of arrhythmia. Several ECG measurement equipments for zebrafish have been developed, and mostly of them consist of electrode or micropipette and electrical filter (Milan et al., 2006; Chaudhari et al., 2013; Dhillon et al., 2013). Detection of 3 dpf larva is also possible (Chi et al., 2008). However, the invasive injury and anesthesia could cause damage to the individual. As for high throughput screening, a fast and stable method of ECG measurement is required, but existing devices seem not compatibility. Computerized recognition of ECGs has become a well-established practice, assisting to classify long-term ECG recordings, which suggests new approaches like Machine Learning are able to recognize and classify the rhythm signal. For instance, automatic classification of single-lead ECG signals with Deep learning (also known as unsupervised feature learning or representation learning) was established (Singh et al., 2018). A new semi-supervised approach based on deep learning and active learning for classification of electrocardiogram signals is proposed (Sayantan et al., 2018). Though several algorithms have focused on automatically classifying heartbeats in ECGs, the scalability failure to handle large intra-class variations wherein the robustness of many existing ECG classification techniques remains limited. We have acquired plenty of dynamic images of different conditions of heartbeat and attempt to establish the relationship between waveform and define characteristic to classify the phenotype. Rhythm classify method based on image processing will be a non-invasive measurement of heart regulation.

In conclusion, we identified 71 compounds from extract of WXKL by LC-HRMS, and firstly utilized a transgenic zebrafish cmlc2-GFP induced by terfenadine as an animal model for screening active compounds from WXKL. After recording heartbeat that affected by fractions under a fluorescent microscope, a convenient image process was applied to exhibit the rhythm of heartbeat. Subsequently, we integrated chemometric analysis with bio-activity *in vivo* model of corresponding fractions and calculated active index of identified compounds. Ginsenoside Rg_1_, ginsenoside Re, notoginsenoside R_1_, lobetyolin, and lobetyolinin were selected to validate activity. Measurement of ECG of adult zebrafish also performed as a complement. Our results suggest that integrate bio-assay and substantial analysis to perform active index calculation improve the efficiency of active compounds discovering from TCM, and this approach is possible to be applied for the research of complex diseases.

## Author contributions

HL and XC designed and performed the experimental work. BZ, KQ, and YS provided the WXKL patent drug and related herb and extract. MB guided the theory of cardiac electrophysiology and pharmacology. All authors proofread the paper and provided feedback.

### Conflict of interest statement

The authors declare that the research was conducted in the absence of any commercial or financial relationships that could be construed as a potential conflict of interest.

## Supplementary material

Supplementary Table 1 Bioactive-coefficient ranking of identified compounds.

Supplementary Figure 1 LC-MS chromatograms of fractions of WXKL. Some fractions were not enough for analysis so were not showed here.

Supplementary Figure 2 Anti-oxidation activity of WXKL fractions. **(A)** Protection rate of all fractions of WXKL, *n* = 3. **(B)** Dose-response of selected fractions that has protective activity.

Supplementary Figure 3 Dose effect of Terfenadine on heart rate of zebrafish. ^**^*P* < 0.01 vs. control. *n* = 8.

Video 1 Side view of heart of zebrafish larva in normal group.

Video 2 Vertical view of heart of zebrafish larva in normal group.

Video 3 Side view of heart of zebrafish larva in terfenadine group.

Video 4 Vertical view of heart of zebrafish larva in terfenadine group.

Video 5 Side view of heart of zebrafish larva in WXKL group.

Video 6 Vertical view of heart of zebrafish larva in WXKL group.
