# Supplementary material for: Screening and Identification of Cardioprotective Compounds From Wenxin Keli by Activity Index Approach and in vivo Zebrafish Model
Source: Front Pharmacol. 2018 Nov 13;9:1288. doi: 10.3389/fphar.2018.01288 (PMC6243390; doi:10.3389/fphar.2018.01288)
Supplement: Supplementary Table 1 — Bioactive-coefficient ranking of identified compounds. [file Table_1.DOCX]

Supplementary Material

Bioactive-coefficient based cardioprotective compounds discovering from Wenxin Keli by in vivo zebrafish model

Hao Liu^1^, Xuechun Chen^1^, Xiaoping Zhao^2*^, Buchang Zhao^3^, Ke Qian^3^, Yang Shi^3^, Mirko Baruscotti^4^, Yi Wang^1*^

*** Correspondence:**

Dr. Yi Wang: mysky@zju.edu.cn;

Dr. Xiaoping Zhao: zhaoxiaoping@zcmu.edu.cn

**Fractions preparation of Wenxin Keli**

100 g granule of Wenxin Keli was dissolved in 1L 70% ethanol and extracted by ultrasound for 1h twice, and the filtrates were merged and followed with decompressing concentration and freeze drying. The concentrated extract was absorbed on D101 microporous resin and eluted with ethanol (5 times of resin volume). The gradient was water, 20% ethanol, 40% ethanol, 95% ethanol, the eluates corresponding Fraction-W, Fraction-1, Fraction-2, Fraction-3. These eluates were concentrated and separated by Semi-preparative HPLC to obtain sub-fractions. Semi-preparative HPLC was conducted on an Agilent 1200 HPLC equipped with a quaternary pump, an auto-sampler, a column compartment and a diode-array (DAD) detector. The Zorbax SB-C18 column (Semi-Preparative, 9.4 mm × 250 mm, 5 μm, Agilent) was used and eluted with the gradient profile of A (water) and B (methanol) at a column temperature of 30 ℃ and at a flow rate of 8.0 mL/min. All eluates were dried by freeze drying.

**Fractions analysis**

Dried fractions were dissolved in methanol and water with final concentration as 2 mg/mL Finnigan LCQ DecaXP^plus^ mass spectrometer equipped with an ESI source (Thermo, MA, USA) coupled to Agilent 1100 liquid chromatography (Agilent, Waldbronn, Germany) was employed. The parameters of LC-MS analysis were as follows: nebulizing gas, high purity nitrogen (N_2_); collosion gas, high-purity helium (He); ion spray voltage: -3 kV; capillary temperature: 350℃; capillary voltage: −15 V; mass range: m/z 100−1500. Chromatographic separation method was the same with the analysis of WXKL.

## Supplementary Figures

**
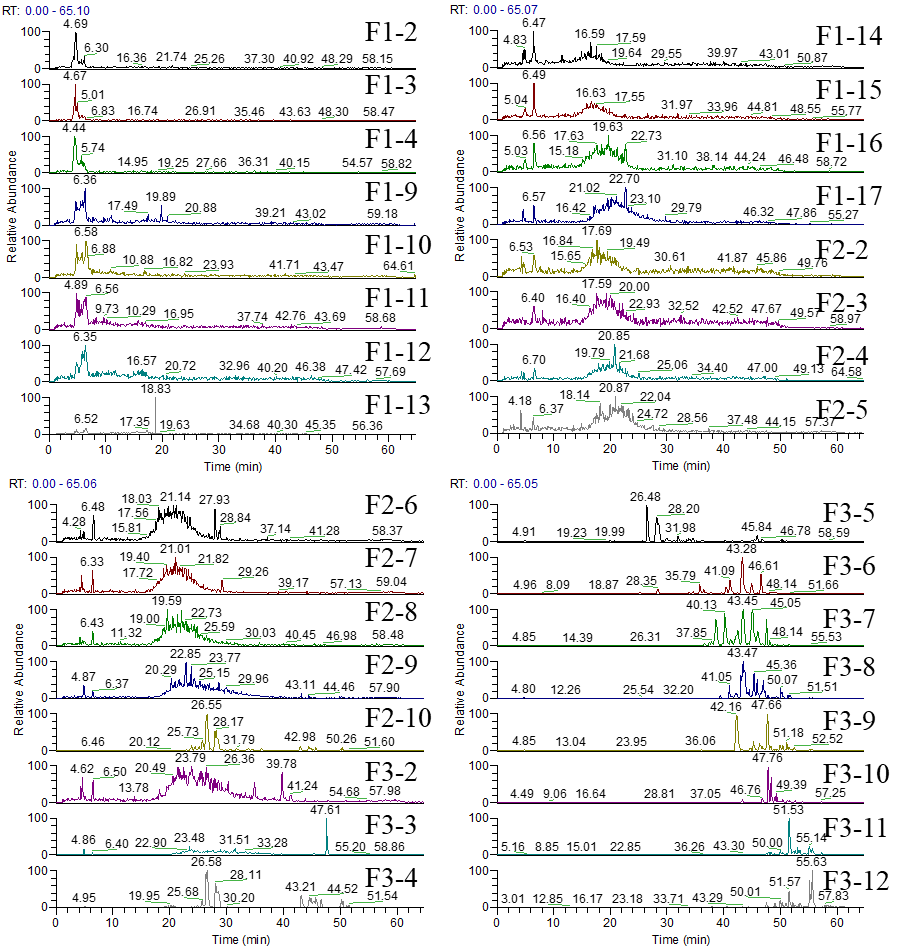
**

**Supplementary Figure 1. LC-MS chromatograms of fractions of WXKL.** Some fractions were not enough for analysis so were not showed here.

**
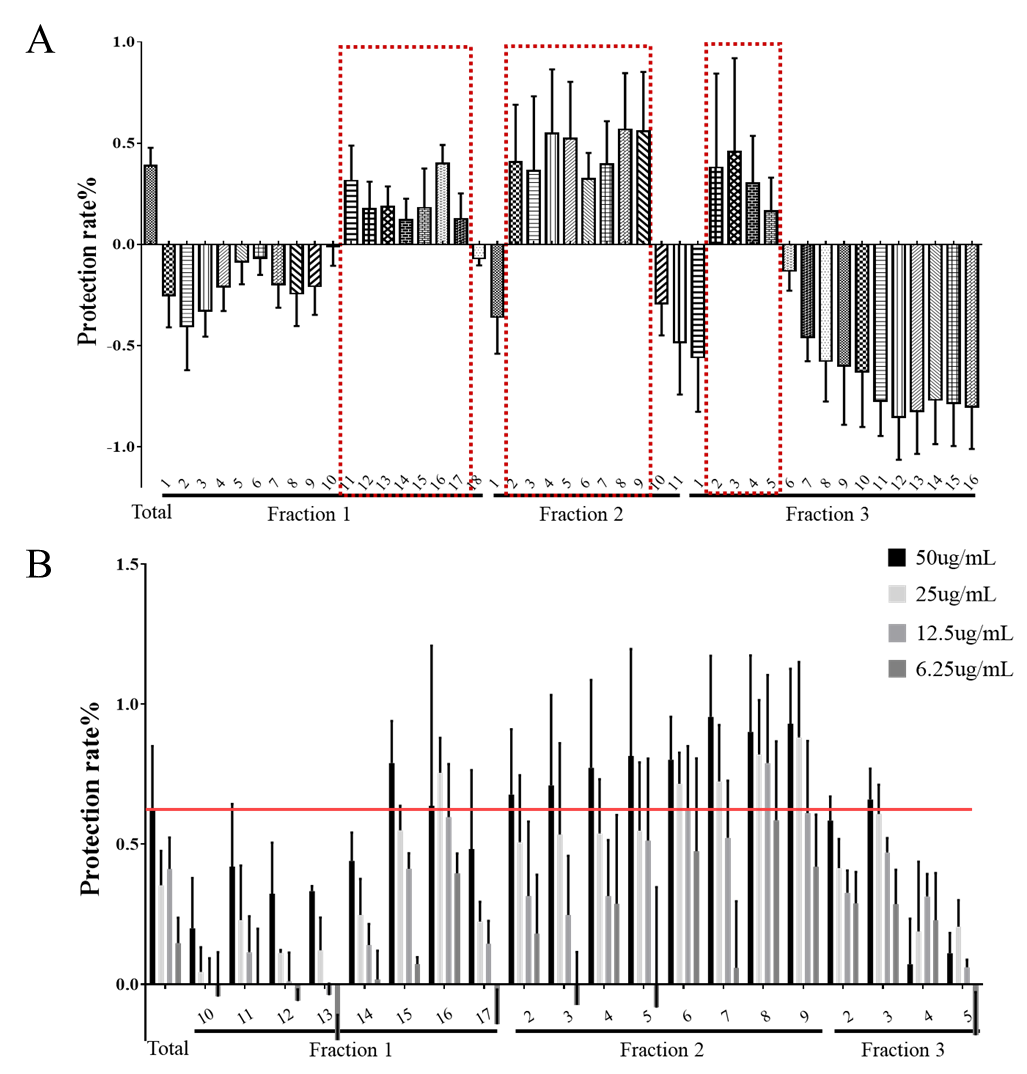
**

**Supplementary Figure 2. Anti-oxidation activity of WXKL fractions.** (A) Protection rate of all fractions of WXKL, n=3. (B) Dose-response of selected fractions that has protective activity.

**
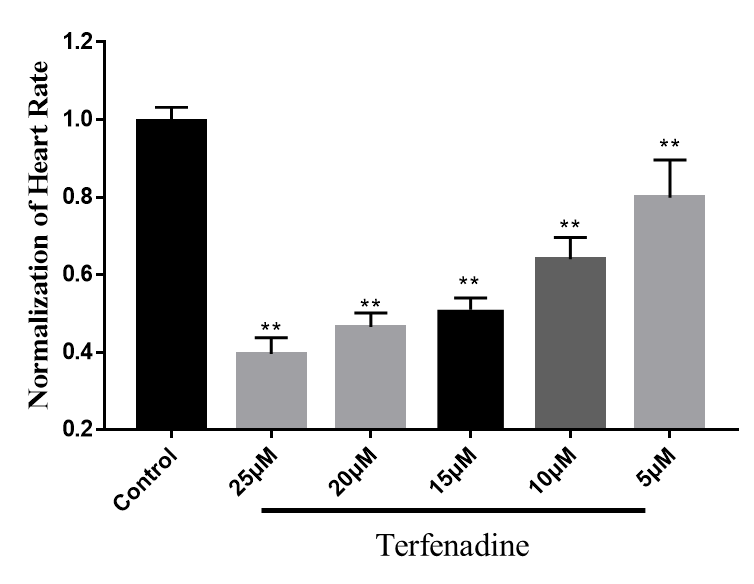
**

**Supplementary Figure 3. Dose effect of Terfenadine on heart rate of zebrafish.** ***P*<0.01 vs Control. n=8.

**Supplementary Table 1. Bioactive-coefficient ranking of identified compounds**

| Identity | Score |
| --- | --- |
| Lobetyolin | 0.24 |
| Echimidine | 0.20 |
| Isoheptanol 2(S)-O-β-D-xylopyranosyl-(1→6)-O-β-D-glucopyranoside | 0.19 |
| Ginsenoside Re | 0.19 |
| Syringin | 0.18 |
| Gentisic acid β-D-glucoside | 0.18 |
| Notoginsenoside G | 0.17 |
| vina Ginsenoside R15 | 0.17 |
| Lobetyolinin | 0.16 |
| Javanicin J | 0.16 |
| Codonopilate A | 0.15 |
| Cipatrijugin G | 0.14 |
| Tangshenoside V | 0.14 |
| Notoginsenoside R1 | 0.13 |
| Difructose anhydride III | 0.11 |
| Hexyl 6-O-beta-D-glucopyranosyl-beta-D-glucopyranoside | 0.11 |
| Vanillic acid 4-O-neohesperidoside | 0.10 |
| Deoxyloganic Acid | 0.10 |
| Neochlorogenic acid | 0.09 |
| 6'-O-(cis-1,4-dihydroxycyclohexanacetyl) acteoside | 0.08 |
| Ginsenoside Rg1 | 0.05 |
| 20-(β-D-glucopyranosyloxy)-ginsenoside Rf | 0.01 |
